# Supplementary material for: Identification of Phytogenic Compounds with Antioxidant Action That Protect Porcine Intestinal Epithelial Cells from Hydrogen Peroxide Induced Oxidative Damage
Source: Antioxidants (Basel). 2022 Oct 28;11(11):2134. doi: 10.3390/antiox11112134 (PMC9687067; doi:10.3390/antiox11112134)
Supplement: Supplementary file 1 [file antioxidants-11-02134-s001.zip › antioxidants-1969840-supplementary.pdf]

Table S1. Primers used in this study.

Gene	Sequence	Product size (bp)	Accession number
GAPDH	F: 5′-GCTACACTGAGGACCAGGTTG-3′<br>R: 5′-CCTGTTGCTGTAGCCAAATTC-3′	146	XM_021091114.1
HO-1	F: 5′-TACCGCTCCCGAATGAACAC-3′<br>R: 5′-GTCACGGGAGTGGAGTCTTG-3′	209	NM_001004027.1
NQO1	F: 5′-GATCATACTGGCCCACTCCG-3′<br>R: 5′-GAGCAGTCTCGGCAGGATAC-3′	200	NM_001159613.1
SOD1	F: 5′-GAGACCTGGGCAATGTGACT-3′<br>R: 5′-CTGCCCAAGTCATCTGGTTT-3′	139	NM_001190422.1
CAT	F: 5′- GGACATGGTCTGGGACTTCT-3′<br>R: 5′- GTCTTGCTGCATCTTCAACG-3′	221	NM_214301.2
GPX1	F: 5′- CCTAGCAGTGCCTAGAGTGC -3′<br>R: 5′- CGCCCATCTCAGGGGATTTT -3′	143	NM_214201.1

Table S2. Antibiotics used in this study.

Antibody	Supplier	Dilution
GAPDH (#REK0005)	Real-Ab (Tianjing, China)	1:5000
Bcl-2 (#4223)	CST (Danvers, MA, USA)	1:500
Bax (cat no:#2772)	CST	1:500
Caspase3 (#9665)	CST	1:500
C-Capase3 (#9664)	CST	1:500
Nrf2 (#A1224)	Abclonal	1:500
p-Nrf2 (#ab76026)	Abcam (Cambrideg, MA, USA)	1:500
Keap1 (#ab118285)	Abcam	1:500
